# Supplementary material for: Influences of study design on the effectiveness of consensus messaging: The case of medicinal cannabis
Source: PLoS One. 2021 Nov 29;16(11):e0260342. doi: 10.1371/journal.pone.0260342 (PMC8629267; doi:10.1371/journal.pone.0260342)
Supplement: S2 Table — (DOCX) [file pone.0260342.s002.docx]

**S2 Table.** Descriptives, *p* values, and Cohen’s *d* for single-sample *t*-tests (pretest/posttest sample)
