# Supplementary material for: Influences of study design on the effectiveness of consensus messaging: The case of medicinal cannabis
Source: PLoS One. 2021 Nov 29;16(11):e0260342. doi: 10.1371/journal.pone.0260342 (PMC8629267; doi:10.1371/journal.pone.0260342)
Supplement: S3 Table — (DOCX) [file pone.0260342.s003.docx]

**S3 Table.** Between Conditions Effects for Posttest Only Sample
